# The Membrane-Proximal Region of C–C Chemokine Receptor Type 5 Participates in the Infection of HIV-1

**DOI:** 10.3389/fimmu.2017.00478

**Published:** 2017-04-24

**Authors:** Yue Tan, Pei Tong, Junyi Wang, Lei Zhao, Jing Li, Yang Yu, Ying-Hua Chen, Ji Wang

**Affiliations:** ^1^Laboratory of Immunology, School of Life Sciences, Beijing Key Laboratory for Protein Therapeutics, Protein Science Laboratory of the Ministry of Education, Tsinghua University, Beijing, China; ^2^The Key Laboratory of Bioorganic Phosphorus Chemistry and Chemical Biology of Ministry of Education, Department of Chemistry, Tsinghua University, Beijing, China; ^3^Wellman Center for Photomedicine, Massachusetts General Hospital, Department of Dermatology, Harvard Medical School, Boston, MA, USA

**Keywords:** C–C chemokine receptor type 5, membrane-proximal region, HIV-1, blocking antibody, HIV vaccine

## Abstract

The initial infection and transmission of HIV-1 requires C–C chemokine receptor type 5 (CCR5). Here, we report that the membrane-proximal region (MPR, aa 22–38) of CCR5 participates in the infection of HIV-1. First, MPR-specific antibodies elicited in mice dose-dependently inhibited the infection of CCR5-tropic HIV-1. Second, substituting MPR with the same region from other co-receptors significantly impaired HIV-1 infection, while the key residues identified by alanine scanning mutagenesis formed an exposed leucine zipper-like structure. Moreover, a peptide derived from MPR could block the infection of a number of HIV-1 strains only before the formation of gp41 six-helix bundle, coincide with the early interaction between CCR5 and the gp120 protein during HIV-1 infection. These promising results ensured the potential of this previously uncharacterized domain as a starting point for the development of antiviral drugs, blocking antibodies, and HIV vaccines.

## Introduction

Identifying novel targets in the infection process of HIV-1 is of great importance for the development of antiviral drugs, antibodies, and vaccines. Multiple targets have been previously identified, including envelop protein, reverse transcriptase, protease, and integrase, etc. (1). However, the small molecule drugs against these well-known targets always induced resistant viruses quickly due to the high mutation rate, which is also a major obstacle for a successful HIV vaccine (2). Targeting the host proteins involved in the viral entry process is another attractive strategy since these proteins are conserved among the human population and unlikely to mutate during the viral infection. The entry of HIV-1 requires two host proteins, the cluster of differentiation 4 (CD4) and the co-receptor. C–C chemokine receptor type 5 (CCR5) and C–X–C chemokine receptor type 4 (CXCR4) are two main co-receptors for HIV-1 infection, though other chemokine receptors including CCR2b and CCR3 may also serve as co-receptors, albeit in rare cases (3). CCR5 is exclusively utilized in the initial infection of HIV-1, whereas CXCR4-tropic strains are rarely observed in the early infection and only become dominant afterward (4). Individuals who carried CCR5Δ32, a naturally existing truncated form of CCR5, showed low susceptivity to HIV-1 infection (5). More importantly, transplanting stem cells homozygous for CCR5Δ32 to an HIV-1 carrier kept this patient from viral rebound (6). This early success indicated that CCR5 could be an exceptional target for anti-HIV-1 therapies, especially for the prevention of initial infection and transmission of HIV-1.

C–C chemokine receptor type 5 belongs to G-protein-coupled receptor family. It consists of an N-terminal domain, three extracellular loops (ECLs), three intracellular loops, and a C-terminal tail (7). N-terminus and ECL2 are two crucial regions involved in the binding between CCR5 and the gp120 protein of HIV-1 (8, 9). The N-terminus binds to the intersection of the four-stranded bridging sheet and the V3 loop on gp120, resulting in rigidification of the V3 stem (10). Then the ECL2 region interacts with the V3 loop and triggers the subsequent conformational changes of gp120 and gp41 protein (11). A number of CCR5 antagonists and monoclonal antibodies (mAbs) hindering these two interactions have been developed to block the viral infection. To date, Maraviroc is the only small-molecule antagonist approved by FDA for clinical treatment and showed potent anti-HIV-1 activity both *in vitro* and *in vivo* (12). 2D7 is one of the most potent mAbs to block HIV-1 entry, recognizing a conformational epitope on ECL2 of CCR5 (13, 14). However, the clinical potential of these inhibitors may be compromised by the rapid establishment of drug resistance (15, 16), as well as the fact that they always blocked the physiological function of CCR5 as a chemokine receptor (17, 18). Actually, these natural signaling pathways of CCR5 played very important role in the innate immunity against several pathogens (19–21). PRO140, another anti-CCR5 mAb targeting a conformational epitope composed of several residues from N-terminus and ECL2, could robustly block the viral entry without interrupting the physiological function of CCR5, indicating the potential of using anti-CCR5 antibodies as anti-HIV reagents (22). In fact, inducing anti-CCR5 antibodies in uninfected individuals to prevent the infection rather than to treat the infection is a more rational approach since the therapeutic effect of inhibitors could be bypassed by the emergence of dual-tropic or CXCR4-tropic viruses (23). Conversely, the initial infection of HIV-1 relies on CCR5 exclusively. Although the initial success has been achieved by inducing antibodies against N-terminus and ECL1 (24–27), these induced antibodies might interfere physiological function of CCR5 by inducing internalization of CCR5 or interfering the ligand binding (28–30). A further understanding on the CCR5-mediated HIV-1 entry and identifying new regions that participate in this process should contribute to a new vaccine design aiming to elicit antibodies that potently block HIV-1 infection without any interference on physiological function of CCR5.

In this study, we find a previously uncharacterized region on CCR5 that is important for the infection of HIV-1. This region locates between the N-terminus and the first transmembrane helix (TM1), designated as the membrane-proximal region (MPR) of CCR5. The antibodies targeting this region block the infection of a CCR5-tropic HIV-1 strain without affecting a CXCR4-tropic strain. Replacing MPR with the corresponding region from CCR3, CCR2b, or CXCR4 significantly abolishes viral infection. The subsequent alanine scanning mutagenesis reveals that I23, N24, and L32 are key residues for HIV-1 infection. Moreover, the peptide derived from this region, modified by conjugating a cholesterol group to the C-terminal to retain its natural location and orientation on the cell membrane, potently inhibits the infection of both lab-adaptive strains and clinical isolates.

## Materials and Methods

### Peptides, Primers, Cells, and Plasmids

The peptides, C17, C17-GSGC, CGSG-C17, and C21 were synthesized by Chinapeptides Corp. (Shanghai, China) with purity >90%. C52L and C34 are kind gifts from Dr. Shibo Jiang at the New York Blood Center (31). The peptide sequences are C17, KINVKQIAARLLPPLYS, C17-GSGC, KINVKQIAARLLPPLYSGSGC, CGSG-C17, CGSGKINVKQIAARLLPPLYS, C21, KINVKQIAARLLPPLYSLVFI, C52L, NHTTWMEWDREINNYTSLIHSLIEESQNLQEKNEQELLELDKWASLWNWFNIKIK, C34, WMEWDREINNYTSLIHSLIEESQNQQEKNEQELL. C17-GSGC and keyhole limpet hemocyanin (KLH) were conjugated to make C17-KLH by Chinapeptides Corp. All primers were synthesized by Sangon Biological Engineering Technology Inc. (Shanghai, China). The plasmids, including pNL4-3.LucR^−^E^−^, pHXB2-Env, pJRFL-Env, and pVSVG, were obtained from the NIH AIDS Research and Reference Reagent Program. The plasmids, including pSF162-Env, pCNE28-Env, and pCNE49-Env, and cell line Ghost-R5 were kindly provided by Dr. Linqi Zhang at Tsinghua University. Ghost-X4 cell is a kind gift from Dr. Zhiwei Wu at Nanjing University.

### Prediction of Antigenic Region

The antigenic region of CCR5 was predicted by three methods and the corresponding online servers, SVMTriP^1^ (32), FBCpred^2^ (33), and PAP^3^ (34).

### Immunization

Three 8-week-old female Balb/c mice were first injected with 100 μg of C17-KLH in complete Freund’s adjuvant (CFA) (Pierce, Rockford, IL, USA) into intraperitoneal cavity at day 0, followed by one boost with a mixture of 100 μg C17-KLH and incomplete Freund’s adjuvant (IFA) (Pierce, Rockford, IL, USA) on day 21, and two boosts with a mixture of 50 μg of C17-KLH and 100 μg of C17 peptide in IFA, on day 35 and day 49. Control group was immunized with PBS in CFA or IFA in priming or boost, respectively. The sera were harvested a week after the final immunization and stored at −20°C.

### Enzyme-Linked Immunosorbent Assay (ELISA)

The immunogenicity of C17 was characterized by indirect ELISA. Microtiter plates were coated at 4°C overnight with 50 µl synthetic peptide C17, KLH-C17 or irrelevant peptide C34 from gp41 protein (all at 5 µg/ml) diluted in NaHCO_3_ (pH 9.6). The wells were then blocked with 0.3% gelatin in PBS at 37°C for 2 h. After wash with PBS containing 0.05% Tween-20, mouse sera diluted in PBS at a fourfold dilution (starting from 1:100) were added, followed by incubation at 37°C for 2 h. The peroxidase-conjugated anti-mouse immunoglobulin antibodies (Dako, Denmark) were used as secondary antibody. After extensive washes, the substrate *o*-phenylenediamine was added and stopped by 2 M H_2_SO_4_ after 10 min. The sera titer was identified as the highest dilution of serum with the average values of 490 nm ≥ 0.2.

### Purification of Total IgGs

The total IgGs (anti-C17) were purified from mouse antisera using protein G affinity column (GE Healthcare) as the manual provided by the manufacturer. Briefly, 1 ml mouse serum was diluted with 4 ml PBS and passed through the affinity column. The column was washed with 20 ml PBS to remove non-specific antibodies. Total IgGs were eluted with low pH elution buffer (pH 2.5, 0.1 M glycine) and neutralized with high pH buffer (pH 8.0, 1 M Tris–HCl). The purified antibodies were then dialyzed against PBS by using a 30 kD ultra centrifugal filter device (Millipore, MA, USA). The antibody concentrations were quantified by measuring absorbance at 280 nm and stored at 4°C until use.

### Flow Cytometry Analysis for Anti-C17 Polyclonal Antibodies

Ghost-R5 cells were incubated with anti-C17 total IgG or normal IgG (purified from negative mouse sera) at a concentration of 20 µg/ml at 37°C for 1 h. After wash with PBS (pH 7.4) containing 2% fetal bovine serum, cells were labeled by Alexa Fluor 647 goat anti-mouse IgG (H + L) (Life Technologies) on ice for 45 min. To demonstrate that the binding was caused by MPR-specific antibodies, anti-C17 was preincubated with 100 µM C17 peptide at 37°C for 1 h. An irrelevant peptide C34 was also used, and 2D7 was used as the positive control. To demonstrate that anti-C17 did not induce the internalization of CCR5, Ghost-R5 cells were preincubation with anti-C17 (100 µg/ml) or medium at 37°C for 2 h, followed by labeling CCR5 with PE-labeled anti-human CCR5 monoclonal antibody (2D7, BD Biosciences, USA). The unlabeled Ghost-R5 cells were used as the negative control. Human peripheral blood mononuclear cells (PBMCs) were also used. PBMCs were first activated with PMA (50 ng/ml, Sigma) and ionomycin (1 µM, Sigma) for 8 h. Activated PBMCs were incubated with anti-C17 (100 µg/ml) or normal IgG for 2 or 16 h, followed by labeling CCR5 with PE-labeled 2D7. The data were collected by BD Accuri C6 (BD Biosciences) and analyzed with FlowJo software.

### Measurement of Cytotoxicity

The XTT assay was used to detect the cytotoxicity of MPR-derived peptides (C21, C17, C17-Chol, and Chol-C17) to Ghost cells as previously described (35). In brief, Ghost cells were plated in 96-well plates (100 μl/well, 1 × 10^5^/ml). Then equal volumes of the peptide at graded concentrations were added. After incubation at 37°C for 4 days, 50 µl of XTT solution (1 mg/ml, containing 0.02 µM phenazine methosulfate) was added. The absorbance at 450 nm was measured after 4 h.

### Calcium Flux Assay

The fluorochromes Fluo-4AM and FuraRed-AM (Life technologies) were added to HEK 293T cells transiently transfected with plasmids expressing wild-type (WT) CCR5 at a final concentration of 1 µM. The cells were washed once after incubation at 37°C for 20 min and resuspended in Hank’s balanced salt solution. Cells (10^6^) were incubated with anti-C17, normal IgG, or 2D7 (BD Pharmingen) at a concentration of 20 µg/ml, followed by stimulation with MIP-1β (1 µg/ml, R&D systems). The data were collected by BD Accuri C6 using time and events model. Calcium level was reflected by the ratio of Fluo-4/FuraRed fluorescence.

### Construction of CCR5 Mutants

Alanine scan mutations were introduced with the Muta-direct™ site-directed mutagenesis kit (SBS Genetech, Beijing, China). In the CCR5-CCR3, CCR5-CCR2b, and CCR5-CXCR4 chimeras, the nucleotides coding for amino acids 22–38 of the hCCR5 receptor were replaced by nucleotides coding for amino acids 26–42 of the hCCR3, 34–50 of the hCCR2b, and 38–54 of the hCXCR4, respectively. Totally 3 µg of WT or mutated CCR5 plasmids were transfected into 293T cells in 6-well plate using transfection reagent VigoFect (Vigorous Biotechnology Beijing Co., Ltd., China) as instructions of the manufacturer. After 24 h, flow cytometry was performed to check the surface protein expression level. After washing with phosphate buffer solution (PBS, pH 7.4) containing 2% fetal bovine serum, the cells were labeled by FITC-conjugated mouse anti-human CCR5 monoclonal antibody (BD Biosciences, USA) at room temperature for 45 min. The data were collected by BD Accuri C6 and analyzed by CFlow Plus software.

### Synthesis of Cholesterol Modified Peptides

Rink Amide HMBA resin was used for the solid-phase peptide synthesis (SPPS) with the loading of 0.23 mmol g^−1^. The SPPS cycles were performed by microwave peptide synthesizer (Liberty, Discovery, CEM Corporation). The scale used for each peptide was 0.1 mmol. The coupling reagent for Fmoc-amino acid building blocks was HATU (1-[bis (dimethylamino) methylene]-1H-1,2,3-triazolo[4,5-b] pyridinium 3-oxid hexafluorophosphate), which was conducted under microwave irradiation for 10 min with a power of 20 W at 50°C. This process was repeated twice when coupled with Fmoc-Arg(pbf)-OH. The amino acids (5 equiv.) and HATU (5 equiv.), which were dissolved in DMF and DIEA (10 equiv.), were then dissolved in NMP. Those ingredients were added to the reacting system automatically. With the synthesis of C17-GSGC-Chol [Gly–Ser–Gly–Cys (GSGC) as a flexible linker], the first building block Fmoc-Asp (OH)-OChol as a side chain anchor was added manually. This building block (4 equiv.) was reacted with the resin in room temperature for 2 h, with HATU (4 equiv.), and DIEA (8 equiv.), which were dissolved in DMF/DCM (1:1) solvent. This side chain anchored reaction was followed by capping reagent treatment, which helped the unreacted amino groups capped by acetylation using HOBt (0.013 m) in Ac2O/DIEA/DMF (4.75:2.25:93.0 v/v/v), and the capping was carried out under microwave irradiation at 65°C for 30 s, and 30 s again with power of 40 W. Piperidine (20%)/HOBT (0.1 m) in DMF was used for Fmoc deprotection, which was under the condition of microwave irradiation at 75°C for 180 s, and 180 s again with a power of 48 W. For Chol-CGSG-C17 peptide, the last cycle with the building block Fmoc-Asp(OH)-OChol was coupled manually, which followed the same procedure with the Fmoc-Asp(OH)-OChol coupling in C17-GSGC-Chol peptide. After finishing all the coupling cycles, the resin was transferred to a flask, followed by a treatment with trifluoroacetic/triisopropylsilane/H_2_O (95:2.5:2.5 v/v/v) cocktail for 2 h in room temperature to detach the peptide from the resin. The peptide detached from the resin was washed by diethylether three times then dried over vacuum. Those impure peptides were purified *via* preparative HPLC with a polar CN column with a flow rate of 7 ml/min. The pure peptide was dried by lyophilizer and was stored in −20°C.

### HIV-1 Pseudovirus Neutralization Assay

Single-cycle pseudovirus infection assay was performed as previously described (36, 37). Briefly, pseudoviruses were packaged by transient transfection of HEK 293T cells with pNL4-3.LucR^−^E^−^ and plasmids expressing Env of HXB2 (B, X4), JRFL (B, R5), SF162 (B, R5), CNE28 (AE, R5), and CNE49 (BC, R5). Pseudovirus expressing vesicular stomatitis virus G (VSVG) protein served as a control. The titer of pseudoviruses was determined by twofold dilution in 1 × 10^4^ Ghost cells to final volume of 200 µl.

To measure capabilities of CCR5 mutants in supporting viral infection, Ghost-X4 cells were transfected with 60 µg of plasmids expressing WT or mutated CCR5 in 100 mm culture dish using VigoFect. Cells were harvested 36 h after transfection and distributed in 96-well plates with 1 × 10^4^ cells/well. After 12 h in 96-well plate, the cells were infected with 200 µl of JRFL or SF162 pseudovirus. The cells were then cultured at 37°C in 5% CO_2_ for 48 h, and the relative luminescence units (RLUs) were measured by a luciferase kit (Promega, USA) and luminometer (Ultra 386, Tecan, USA). Results were normalized to the reporter activity measured with WT CCR5.

To evaluate the antiviral effects of peptides or antibodies, 1 × 10^4^ of Ghost cells were plated into 96-well plates 12 h before HIV-1 pseudovirus infection. For inhibitory activities of anti-C17, 100 µl of anti-C17 at a fourfold serial dilution from 5 µM was incubated with Ghost cells at 37°C for 30 min. Then 100 µl of JRFL, HXB2, CNE28, or CNE49 pseudovirus was added. To neutralize MPR-specific antibodies, anti-C17 was preincubated with 25 µM C17 peptide for 1 h. For inhibitory activities of MPR-derived peptides, 50 µl of MPR-derived peptides (C17, C17-Chol, or Chol-C17) at a twofold serial dilution from 50 µM was incubated with 1 × 10^4^ of Ghost cells at 37°C for 30 min. Then equal volume of JRFL, SF162, CNE28, CNE49, or VSVG pseudovirus was added to the mixture. For the time course experiment, 50 µl of JRFL pseudovirus was added to 100 µl of Ghost-R5 cells (0 min). Fifty microliters of C17-Chol peptide (final concentration 10 µM) or C52L peptide (final concentration 100 nM) were added to Ghost-R5 cells and JRFL virus mixture at time 0, 10, 20, 40, and 60 min. The cells were cultured at 37°C in 5% CO_2_ and lysed 48 h later for RLU detection. RLUs were detected as the aforementioned methods.

### Statistical Analysis

Two tailed *t*-test (*t*-test) was used to analyze the difference between two groups. *p* Value was calculated by PRISM software (GraphPad, CA, USA), and a difference is regarded significant if *p* is less than 0.05.

## Results

### Antibodies Targeting the MPR of CCR5 Blocked HIV-1 Infection

**Figure 1:**
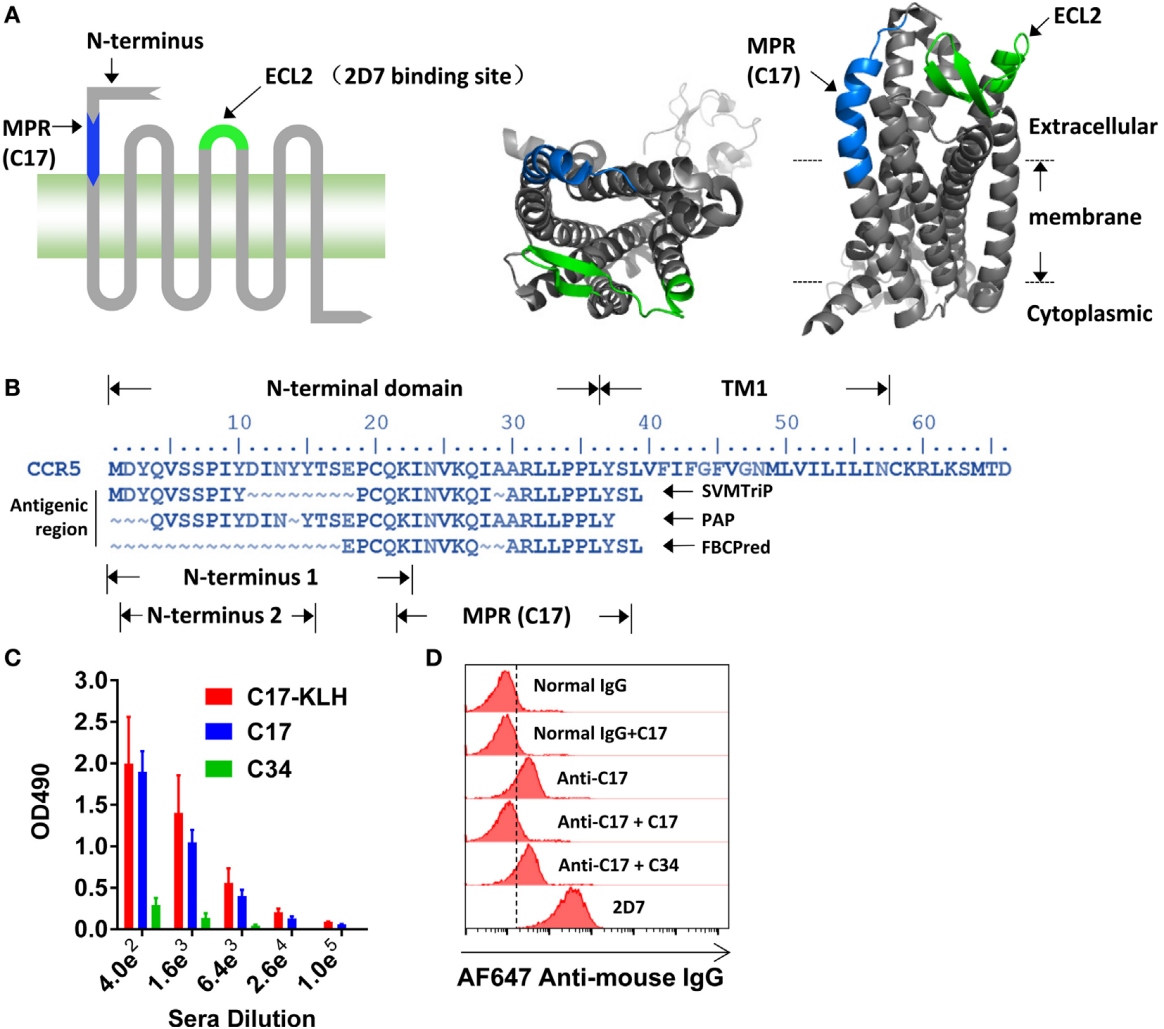
**Inducing membrane-proximal region (MPR)-specific antibodies in mice**. **(A)** C–C chemokine receptor type 5 (CCR5) consists of an N-terminal, three extracellular loops (ECLs), three intracellular loops, and a C-terminal tail. The structure of CCR5 was rendered by Pymol software based on a previously reported structure 4MBS (7). MPR and ECL2 are highlighted in blue and green, respectively. ECL2 contains a binding site for 2D7. **(B)** The amino acid sequence of N-terminal domain and first transmembrane helix (TM1) of CCR5. The antigenic regions were predicted by SVMTrip, PAP, and FBCPred methods and shown under the sequence of CCR5. **(C)** Balb/c mice were immunized with one dose of C17-keyhole limpet hemocyanin (KLH) plus complete Freund’s adjuvant, one dose of C17-KLH plus incomplete Freund’s adjuvant (IFA), and two doses of a mixture of C17-KLH, C17 peptide, and IFA. Sera were collected 1 week after the last immunization and antibody titers against C17-KLH, C17, or an irrelevant C34 peptide were measured by enzyme-linked immunosorbent assay. *n* = 3. **(D)** Total IgGs were purified from antiserum, designated as anti-C17. The binding of anti-C17 to CCR5 expressed on the cell surface of Ghost-R5 cells was determined by flow cytometry. Normal IgGs purified from the sera of un-immunized mice served as a negative control, while 2D7 served as a positive control. Alexa Fluor 647-conjugated anti-mouse IgG was used as the secondary antibody. C17 peptide and an irrelevant C34 peptide were used to confirm the binding specificity. Data are presented as mean ± SEM. All experiments were repeated twice with similar results.

The N-terminus and ECL2, illustrated in Figure 1A, has been demonstrated to be important for the entry of HIV-1, whereas the potential role of other domains on CCR5 has not been well documented. Antibody is a well-accepted tool to study the function of a specific region. A set of antibodies, including 2D7, have been used to demonstrate the crucial role of N-terminus and ECL2 in the cellular entry of HIV-1 (17). By using bioinformatic methods, including SVMTrip (32), FBCPred (33), and PAP (34), to identify potential antigenic regions on CCR5, we found a previously unnoticed region located between N-terminus and TM1 (Figure 1B). A 22 amino acid (aa)-length region (N-terminus 1 in Figure 1B) has been reported to be important for HIV-1 infection (9, 38), and the key region of N-terminus was subsequently shortened to 14 aa in length (N-terminus 2 in Figure 1B) (10). To minimize any potential spatial and functional overlap with previously well-characterized N-terminus, we selected a 17 aa length region that only shared one lysine (K22) residue with N-terminus 1. Because this region located closely to the membrane (Figure 1A, right panel), we named it as MPR, while the peptide derived from this region was designated as C17 (Figure 1B). The K22 was kept to improve the solubility of C17.

Direct blocking of MPR by antibodies was taken to study the role of MPR in HIV-1 infection. To generate antibodies against MPR, the C17 peptide was conjugated to KLH by a flexible linker GSGC. Balb/c mice were primed by intraperitoneal injections of 100 µg C17-KLH with CFA, followed by one booster with a mixture of 100 µg C17-KLH with IFA, and then two boosters with a mixture of 50 µg C17-KLH, 100 µg C17 peptide and IFA. This immunization strategy elicited relatively high IgG titers (geometric mean = 1:25,600) against both C17-KLH and C17 peptide, but not an irrelevant peptide C34 (Figure 1C). For further function studies, the total IgGs were purified from antisera by protein G affinity column to remove any non-specific inhibitory effect of serum components. These purified antibodies were designated as anti-C17 in following functional studies. As demonstrated by flow cytometry analysis, anti-C17 not only bound to peptides coated on an assay plate but also bound to cells expressing natural CCR5 proteins (Figure 1D). This binding could be blocked by preincubating anti-C17 with C17 peptide, confirming the specificity of the binding (Figure 1D). In contrast, preincubating anti-C17 with an irrelevant peptide C34 did not block the binding (Figure 1D).

**Figure 2:**
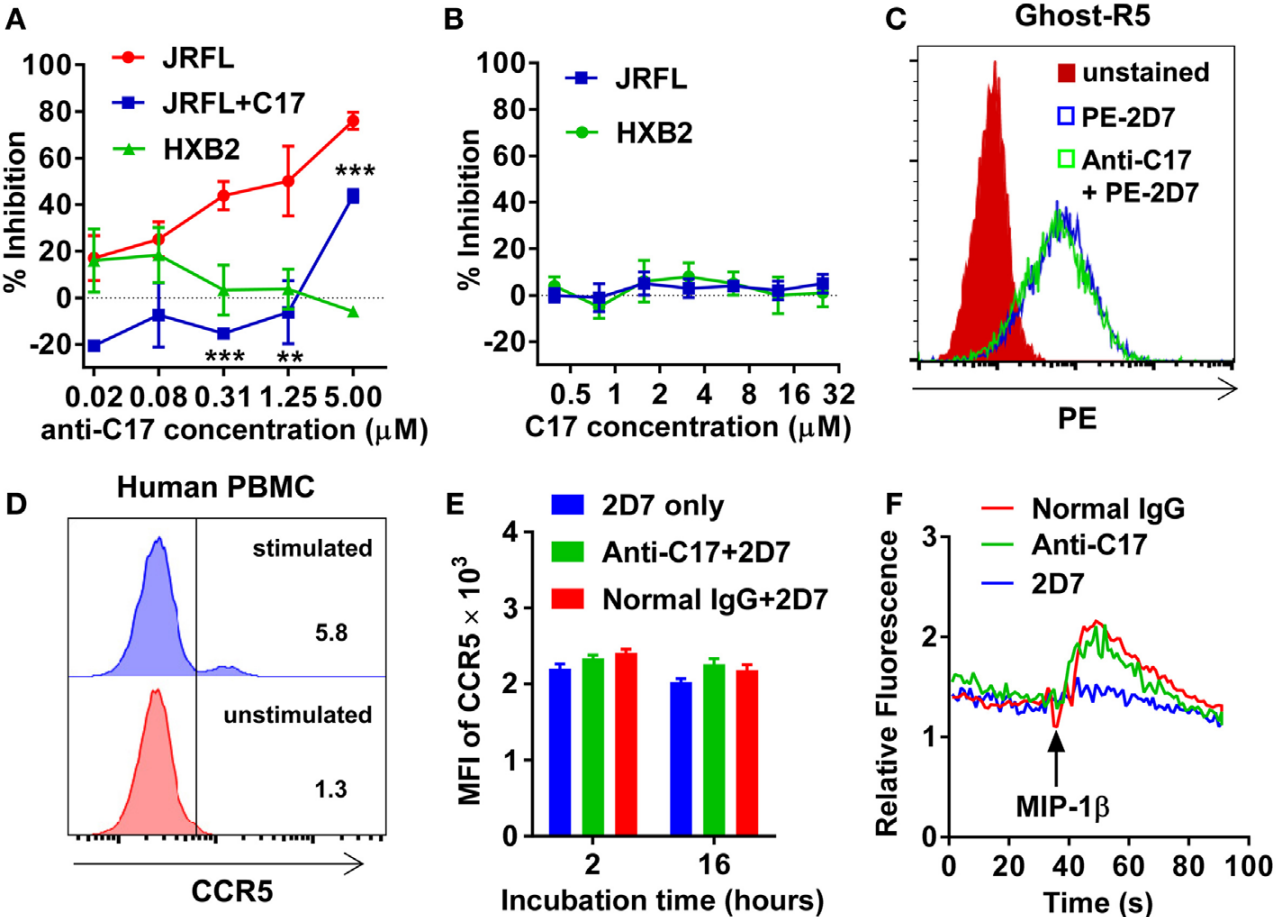
**Membrane-proximal region (MPR)-specific antibodies inhibit HIV-1 infection**. **(A)** Ghost-R5 or Ghost-X4 cells were incubated with fourfold serially diluted anti-C17, followed by infection of JRFL or HXB2 pseudovirus, respectively. Anti-C17 was preincubated with 25 µM C17 peptide to neutralize MPR-specific antibodies, and tested for the inhibition of JRFL pseudovirus (JRFL-C17). *n* = 3. An unpaired *t*-test was performed to show the difference between JRFL and JRFL-C17 at each concentration. **(B)** Ghost cells were incubated with twofold serially diluted C17 peptide, followed by infection of JRFL or HXB2 pseudovirus. *n* = 3. **(C)** Ghost-R5 cells were preincubated with anti-C17 at 37°C for 2 h. The C–C chemokine receptor type 5 (CCR5) on the cell surface was stained by PE-conjugated 2D7 antibody, followed by flow cytometry analysis. **(D)** Human peripheral blood mononuclear cells (PBMCs) were activated by PMA and ionomycin. CCR5^+^ cells were stained by 2D7 and gated. **(E)** Activated PBMCs were incubated with anti-C17 for 2 or 16 h, and mean fluorescence intensity (MFI) of 2D7-labeled CCR5 on CCR5^+^ cells was summarized. *n* = 3. **(F)** 293T cells expressing CCR5 were first incubated with antibodies, followed by stimulation with MIP-1β. The calcium flux indicated by fluorescence ratio of Fluo-4/FuraRed was recorded by flow cytometry. Data are presented as mean ± SEM. All experiments were repeated twice with similar results (**p < 0.01; ***p < 0.001).

Then, the antiviral effect of antibodies was evaluated by pseudovirus expressing envelop proteins from various HIV-1 strains. The strain name was used to represent individual pseudovirus hereafter unless indicated particularly. Anti-C17 could dose dependently inhibit the infection of CCR5-tropic JRFL virus at an IC_50_ of 0.40 µM without any inhibition on CXCR4-tropic HXB2 virus (Figure 2A; Table 1). Furthermore, if anti-C17 was preincubated with C17 peptide to neutralize MPR-specific antibodies, the inhibitory capability of anti-C17 was completely lost at most antibody concentrations tested (Figure 2A). The impaired inhibitory capability was not caused by the direct blockage of viral infection by C17 peptide, because the peptide alone neither inhibited nor enhanced the infection of JRFL and HXB2 (Figure 2B). At the highest concentration (5 µM) of anti-C17, the blocking capability was still impaired significantly (*p* < 0.001) but not completely, probably due to the insufficient amount of C17 peptide to neutralize all C17 binding antibodies (Figure 2A). These results demonstrated that the inhibition was caused exclusively by antibodies targeting the MPR region. Moreover, the inhibition effect of anti-C17 was also confirmed in two clinic isolated strain CNE28 and CNE49 albeit with a higher IC_50_ compared to JRFL (Table 1). Since some antibodies against ECL1 or N-terminus inhibited the viral infection by inducing the internalization of CCR5 (27, 39), the potential effect of anti-C17 on the receptor internalization was further evaluated. In sharp contrast to antibodies against ECL1, MPR-specific antibodies were less likely to induce the internalization of CCR5 receptor because incubating anti-C17 with Ghost-R5 cells at 37°C for 2 h did not decrease the amount of cell surface CCR5 labeled by PE-conjugated 2D7 antibody (Figure 2C), whose epitope located far from MPR (Figure 1A). Human PBMCs were enrolled to further confirm this result in cells with physiological CCR5 density. PBMCs were first activated and a small amount of cells became CCR5+ (Figure 2D). Incubating anti-C17 with these cells for either 2 or 16 h did not reduce the mean fluorescence intensity (MFI) of 2D7-labeled CCR5 (Figure 2E). Therefore, the inhibitory effect of anti-C17 was not ascribed to the internalization of CCR5. Next, the potential effect of anti-C17 on the physiological function of CCR5 was assessed by a calcium flux assay. As shown in Figure 2F, anti-C17 did not inhibit the calcium influx induced by MIP-1β, whereas 2D7 greatly abolished the function of CCR5. Taken together, the inhibition of HIV-1 infection by antibodies against MPR region suggested the important role of MPR in viral infection.

**Table 1:** **Inhibition of HIV-1 pseudovirus infection by anti-C17**.

Strain	Clade	Co-receptor	IC_50_ (μM)
JRFL	B	C–C chemokine receptor type 5 (CCR5)	0.40 ± 0.19
CNE28	AE	CCR5	0.70 ± 0.15
CNE49	BC	CCR5	0.88 ± 0.27
HXB2	B	C–X–C chemokine receptor type 4	>5

### Substituting of MPR in CCR5 Resulted in Impaired HIV-1 Infection

**Figure 3:**
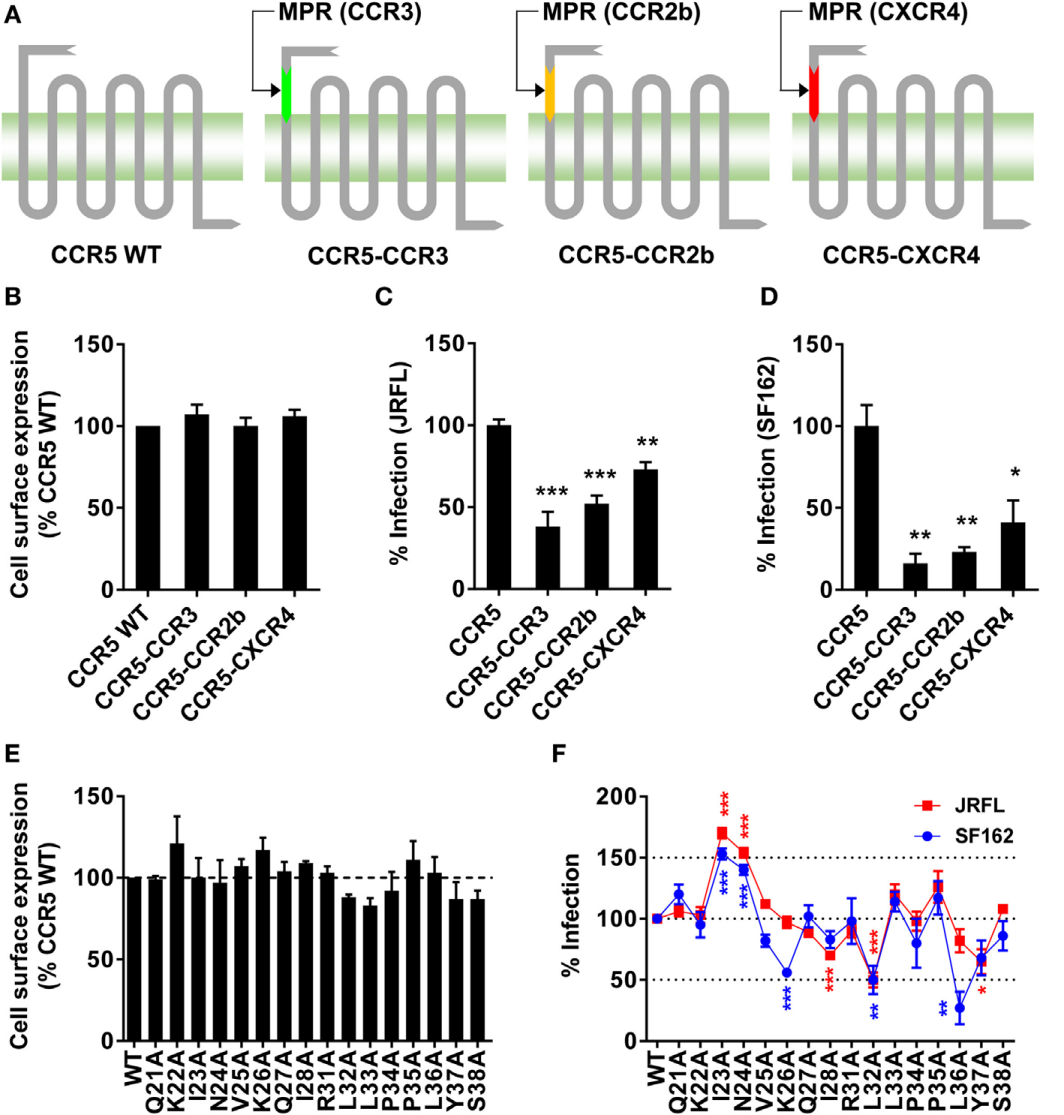
**Substitution or single-residue mutation of membrane-proximal region (MPR) affected HIV-1 infection**. **(A)** A schematic diagram of C–C chemokine receptor type 5 (CCR5) chimeras. The MPR domain of CCR5 was replaced with the sequence from the same domain of other co-receptors to generate three chimeras, CCR5–CCR3, CCR5–CCR2b, and CCR5–C–X–C chemokine receptor type 4 (CXCR4). **(B)** HEK 293T cells were transiently transfected with plasmids expressing wild-type (WT) CCR5 or chimeras, followed by flow cytometry analysis to measure the expression. *n* = 3. **(C,D)** Ghost cells expressing human cluster of differentiation 4 (CD4) but not CCR5 were transiently transfected with plasmids expressing WT CCR5 or chimeras, followed by infection with JRFL **(C)** or SF162 **(D)** pseudovirus. *n* = 4–5. **(E)** HEK 293T cells transiently transfected with WT or mutated CCR5 plasmids were measured for CCR5 expression by flow cytometry. *n* = 3. **(F)** Ghost cells expressing human CD4 but not CCR5 were transiently transfected with plasmids expressing WT or mutated CCR5, followed by the infection with JRFL (red) or SF162 (blue). *n* = 4–10. An unpaired *t*-test was performed to show the difference between WT and mutants. Data are presented as mean ± SEM. All experiments were repeated three times with similar results (*p < 0.05; **p < 0.01; ***p < 0.001).

The antibody blocking experiment could not fully rule out the possibility that MPR-binding antibodies might interrupt the interaction between N-terminus and gp120 by the steric hindrance. To give another line of evidence showing MPR did play roles in HIV-1 infection, we replaced MPR of CCR5 with the same region from other three co-receptors (Figure 3A). The chimeras showed similar surface expressing level as WT CCR5, demonstrated by flow cytometry analysis after transient transfection (Figure 3B). Two commonly used CCR5-tropic strains, JRFL and SF162, were used to check whether these CCR5 chimeras could still support viral entry. As shown in Figure 3C, CCR5–CCR3 and CCR5–CCR2b chimeras, expressed in Ghost cells carrying human CD4 but not CCR5, showed significantly (*p* < 0.001) impaired capability to support JRFL infection, achieving less than 50% efficacy of WT CCR5. CCR5–CXCR4 also significantly (*p* < 0.01) impaired the viral infection, albeit to a lesser extent (Figure 3C). The infection of SF162 strain was more sensitive to the substitution of MPR, since all chimeras showed very poor capability to support the infection of SF162 (Figure 3D).

### Identifying Key Residues in MPR for HIV-1 Infection

The alanine scanning mutagenesis was then performed to identify key residues participating in the viral infection. We found none of the single-residue mutations obviously (>125% or <75%) affected the expression level of CCR5 on the cell surface (Figure 3E). Then the Ghost cells carrying individual mutation were infected by JRFL or SF162 virus. As shown in Figure 3F, some of the mutations significantly enhanced or impaired the viral infection. For JRFL strain, I28A, L32A, and Y37A mutations impaired the viral infection by 30–50%, whereas I23A and N24A enhanced the infection by more than 50%. The importance of MPR was also confirmed in SF162 strain. Similar to JRFL, I23A, and N24A mutations consistently enhanced the viral infection by approximately 50%, while L32A mutation impaired the infection by 50% (Figure 3F). Slightly different from JRFL, two non-significant mutations for JRFL, K26A, and L36A, greatly impaired the viral infection by 50 or 75%, respectively, indicating SF162 strain was more sensitive to CCR5 MPR mutations (Figure 3F), consistent with the results from the MPR substitution experiment (Figure 3D). Although there was a slight difference between JRFL and SF162 in the alanine scanning mutagenesis, the key residues including I23, N24, and L32 which shared similar and obvious effects on both strains confirmed the role of CCR5 MPR in HIV-1 infection.

**Figure 4:**
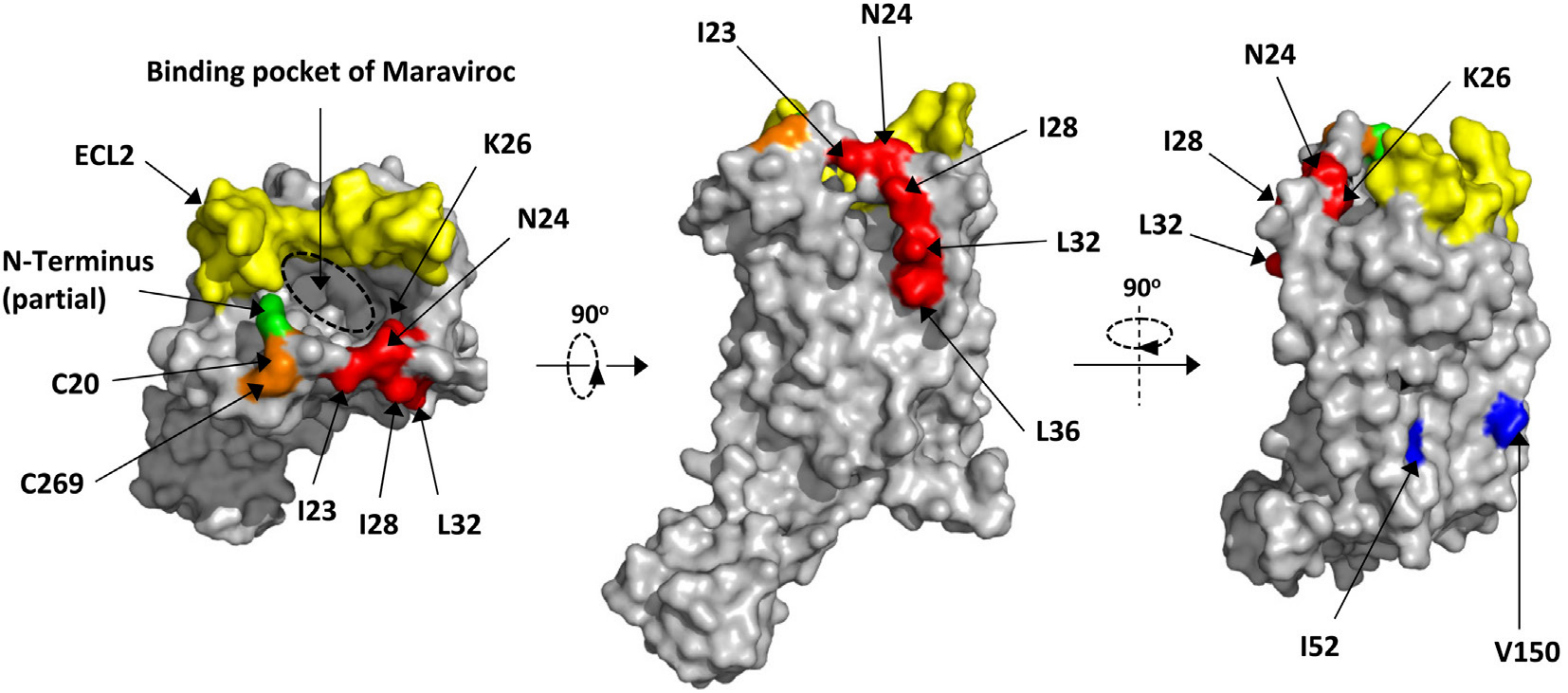
**Location of key residues on the structure of C–C chemokine receptor type 5 (CCR5)**. The key residues were highlighted in red on the structure of CCR5. The surface was generated by Pymol based on 4MBS (7). The binding pocket of Maraviroc was indicated by dashed circle. The key residues for dimerization were highlighted in blue. The partial N-terminus was highlighted in green, while the ECL2 and disulfide bond were in yellow and orange, respectively.

Next, we mapped all key residues (highlighted in red) that significantly affected the infection of either JRFL or SF162 in the structure of CCR5 (Figure 4) (7). Surprisingly, the residues I23, I28, L32, and L36 were all located outside the protein and formed a leucine zipper-like structure (Figure 4, middle panel). Clearly the function of this leucine zipper-like structure was not to mediate the dimerization of CCR5, since two key residues (highlighted in blue) for dimerization of CCR5 located on a different face of the protein (Figure 4, right panel) (40). This leucine-zipper-like structure was separated from the highly flexible N-terminus (green) by a disulfide bond (orange) and located away from ECL2 (yellow), which formed the binding site of natural CCR5 ligands with N-terminus (Figure 4, left panel). Moreover, the leucine zipper faced to the outer space of CCR5 rather than the binding pocket of Maraviroc, an antagonist of CCR5, indicating that this structure may not contribute to the binding of CCR5 ligand (Figure 4, left panel).

### The MPR-Derived Peptide Inhibited HIV-1 Infection

The altered capability of CCR5 chimeras or mutated CCR5 in supporting viral infection could be ascribed to the conformational change of other parts in CCR5 or the interruption of the interaction between MPR and viral proteins. If the interaction is the reason, the peptide derived from MPR may be able to bind to the corresponding domain on viral proteins and affect the viral infection. In fact, peptide inhibition has been extensively used to study the interaction between CCR5 and viral proteins (38, 41, 42).

**Figure 5:**
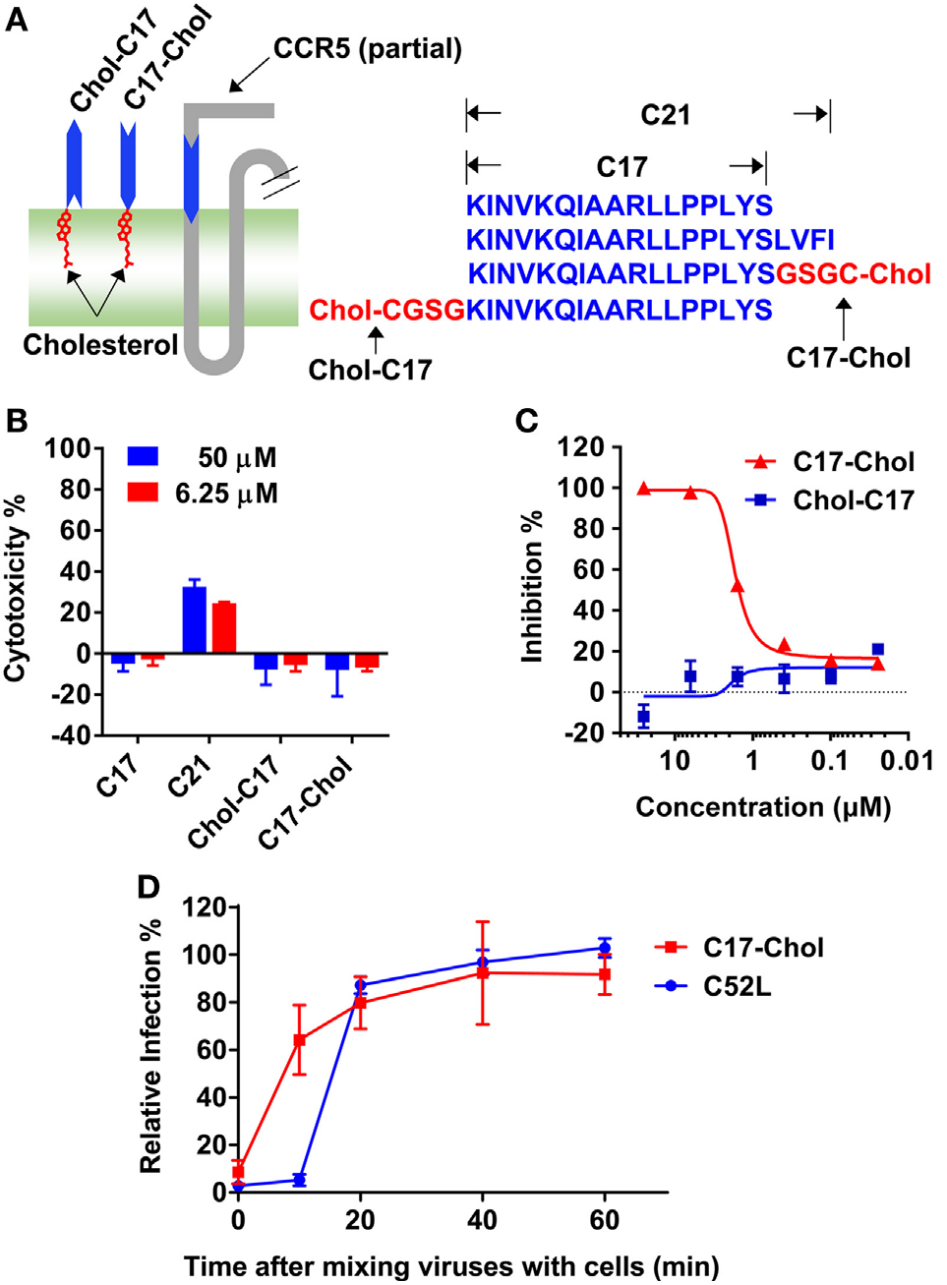
**C17-Chol inhibited the infection of HIV-1**. **(A)** A schematic representation of C17-Chol, Chol-C17, and a part of C–C chemokine receptor type 5 (CCR5). C17-Chol mimics the natural orientation of membrane-proximal region on CCR5 (left). The sequence of C17 is shown in blue, while the linker and cholesterol are highlighted in red (left). C21 peptide has four additional residues from first transmembrane helix. **(B)** Cytotoxicity of C17 and its derivatives in Ghost cells were measured by XTT assay. *n* = 6. **(C)** Ghost-R5 cells were incubated with C17-Chol or Chol-C17, followed by the infection of SF162 pseudovirus. *n* = 3. **(D)** Ghost-R5 cells were infected by JRFL pseudovirus. C17-Chol (10 µM) or C52L (100 nM) was added at various time points after the mixing of cell and viruses. *n* = 3–4. Data are presented as mean ± SEM. All experiments were repeated twice with similar results.

Unexpectedly, C17 peptide showed no inhibitory effect (IC_50_ > 50 µM) on all strains tested (Table 2), probably due to insufficient time for them to arrive at the target (43). One promising way to break this limitation was concentrating peptides at the place where the interaction happens. In this case, anchoring C17 to the cell membrane might enhance its inhibitory activity, since HIV-1 entry happened on the lipid raft and MPR was located closely to the membrane. Additionally, membrane anchoring could also constrain the peptide to the right orientation rather than approaching the target randomly (Figure 5A). Enhancing the membrane anchoring by extending the peptide to the transmembrane domain was not a good strategy, since C21 peptide containing additional four residues from TM1 showed obvious cytotoxicity which was very likely to give false positive results (Figure 5B). Therefore, we used an alternative strategy by conjugating cholesterol to C17. Cholesterol was an ideal membrane anchor to enrich the peptide on the cell surface *via* interacting with lipid rafts (43, 44). We designed two derivatives of C17 with cholesterol conjugated to the N- or C-terminal of the C17 peptide (Chol-C17 or C17-Chol) as shown in Figure 5A. C17-Chol was expected to mimic the physiological location and orientation of MPR, since it is the C-terminal of C17 located more closely to the cell membrane (Figure 5A). On the other hand, Chol-C17, whose C17 bound to membrane in an inappropriate orientation, was synthesized as a control to rule out any non-specific inhibitory effect caused by cholesterol conjugation. To synthesize cholesterol conjugated peptides, a linker (GSGC or CGSG) was added to the C-terminal or N-terminal of C17 to provide a conjugating site for cholesterol (Figure 5A, right panel). Both cholesterol-conjugated peptides showed no cytotoxicity at high (50 µM) or low (6.25 µM) concentration (Figure 5B).

**Table 2:** **Inhibition of HIV-1 pseudovirus infection by membrane-proximal region derived peptides**.

Strain	Clade	C17		C17-Chol	
		IC_50_ (μM)	IC_90_ (μM)	IC_50_ (μM)	IC_90_ (μM)
SF162	B	>50	>50	1.04 ± 0.10	4.22 ± 0.54
JRFL	B	>50	>50	1.38 ± 0.68	9.18 ± 1.99
CNE28	AE	>50	>50	0.55 ± 0.06	3.30 ± 0.16
CNE49	BC	>50	>50	0.42 ± 0.06	2.24 ± 0.19
Vesicular stomatitis virus G	N/A	>50	>50	>50	>50

Next, the inhibitory effects of two conjugates were measured. As expected, C17-Chol inhibited the infection of SF162 at a low micromolar level (IC_50_ = 1.04 ± 0.10 µM) (Figure 5C; Table 2). In contrast, Chol-C17 showed no inhibitory effect, indicating that conjugating of cholesterol did not cause non-specific inhibition (Figure 5C). This result also demonstrated that the orientation of C17 was crucial for its functionality. Additional HIV-1 strains were also enrolled to evaluate the inhibitory capability of C17-Chol. In sharp contrast to C17 which showed no inhibitory effect (IC_50_ > 50 µM) on all strains tested, C17-Chol inhibited the infection of another lab-adaptive strain JRFL (IC_50_ = 1.38 ± 0.68 µM) and two clinic-isolated strain CNE28 (IC_50_ = 0.55 ± 0.06 µM) and CNE49 (IC_50_ = 0.42 ± 0.06 µM) (Table 2). Moreover, C17-Chol did not inhibit the infection of pseudovirus carrying VSVG protein whose entry did not depend on CCR5, further ruling out the possibility of non-specific inhibitory effects caused by C17-Chol (Table 2).

To further confirm that C17-Chol specifically interrupted the interaction between CCR5 and viral proteins, we next added C17-Chol at different time point after mixing JRFL virus with Ghost-CCR5 cells. As shown in Figure 5D, the inhibitory effect of C17-Chol was greatly impaired if being added as soon as 10 min after the mixing. In contrast, C52L, an inhibitor of gp41 fusion complex formation, could still inhibit viral entry at this time point, but completely lost the inhibitory effect 20 min after mixing virus and cells. These results revealed that C17-Chol targeted to a very early event in the viral infection, consistent with the fact that the interaction between CCR5 and gp120 happened before the formation of gp41 fusion complex (45, 46).

## Discussion

In past decades, tremendous efforts have been taken to understand details of HIV-1 entry, and a number of inhibitors have been developed accordingly, aiming to a cure for HIV-1 infection. Although several entry inhibitors, including Enfuvirtide (T-20) and Maraviroc (12, 47, 48), have been approved and used in the clinic practice, the rapid emerging of resistant HIV-1 strains indicates that identifying new targets for the development of entry inhibitors is still of great importance. In the current study, we provided several lines of evidence to demonstrate that the MPR of CCR5 participated in the infection of HIV-1. First, blocking MPR by antibodies significantly inhibited the infection of HIV-1. Second, I23A, N24A, and L32A mutations within MPR either enhanced or impaired viral infection by more than 50%, and substituting the MPR of CCR5 with the same region from other co-receptors significantly impaired CCR5’s capability to support viral infection. Third, membrane-anchored peptides derived from MPR potently inhibited the viral infection probably by interrupting the interaction between MPR and viral proteins.

Using synthetic peptides derived from CCR5 in biochemical and functional assays is a common approach to explore the molecular details of co-receptor–Env interactions. A sulfated peptide derived from N-terminus was reported to block HIV-1 entry at an IC_90_ ~ 100–200 µM, confirming the interaction between this region and gp120 (38). A similar approach was used to delineate the important role of ECL2 during HIV entry by evaluating the inhibitory capabilities of various peptides derived from ECL2 (42). These ECL2-derived peptides showed an IC_50_ value of 20–600 µM. In our study, the membrane-anchored C17-Chol peptide, derived from MPR of CCR5, showed relatively potent inhibitory capability with an IC_50_ of 0.42–1.38 µM and an IC_90_ of 2.24–9.18 µM. To further strength this result, most non-specific inhibitions have been excluded in our study by the fact that C17-Chol only specifically inhibited the infection of pseudoviruses carrying HIV-1 Env but not VSVG, and conjugating cholesterol to the N-terminal of C17 peptide could not inhibit HIV-1 infection due to the inappropriate orientation of the peptide on cell membrane. In line with our results, other groups also reported that anchoring peptides to cell membrane could greatly enhance their inhibitory effect, given that the natural orientations were well retained (44, 49). Although it is difficult to compare the inhibitory effect of C17-Chol with peptides derived from N-terminus and ECL2 due to the use of different viral assays, the significant inhibitory capability of this MPR-derived peptide still provided important evidence for the direct interaction between MPR and HIV-1 envelop protein.

One interesting finding of this study is the key residues in MPR, including I23, N24, L32 which affected both JRFL and SF162 infection, as well as I28 and L36 which affected one of the strains, formed a leucine zipper-like structure (Figure 4). Previous studies did not found this structure since they focused on charged residues like K22, K26, and R31 whose alanine substitution did not impair the viral infection except a difference on K22, probably due to the use of different viral strains (8, 9). Our current study also confirmed that these positively charged residues were not important for the cellular entry of HIV-1 (Figure 3F). Actually these Leucine zipper-like structures played important role in protein–protein interactions during cell signaling as well as viral infection (50, 51). For instance, the N-heptad repeat and C-heptad repeat of HIV-1 gp41 protein were zipped together by multiple interactions between leucine, isoleucine, and valine to form a six-helix bundle, facilitating the membrane fusion (52, 53). Because the leucine zipper-like structure always interacted with another leucine zipper-like structure, we speculate that the unknown MPR-binding site on HIV-1 envelop protein may also contains a similar leucine zipper-like structure or a domain with several hydrophobic side chains. This binding site may hide inside the protein complex but transiently expose during the infection according to our peptide inhibition results (Figure 5D). Identifying this binding site on the viral protein will be an important supplement to our understanding of viral entry, inspiring the development of novel antiviral drugs and vaccines.

A prophylactic HIV-1 vaccine remains the ultimate goal for HIV-1 research. Unlike conventional vaccine strategy focusing on highly variable viral proteins, the strategy aiming to induce antibodies against highly conserved CCR5 is an attractive mean. Naturally occurred, HIV-blocking anti-CCR5 antibodies in healthy donors indicated that eliciting the humoral immune responses against CCR5 was feasible (54). The natural-occurred antibodies against N-terminus, ECL1, and ECL2 have been isolated from HIV-exposed, seronegative and long-term non-progressing individuals, and were suggested to be involved in preventing or controlling the viral infection (28, 55–57). Moreover, anti-N-terminus, ECL1 and ECL2 antibodies with HIV-blocking activity have been successfully induced in animals (24–27). Our study contributes to this attractive vaccination strategy by providing a new candidate for the vaccine design. Compare to N-terminus, ECL1 and ECL2, MPR has several unique features. First, unlike ECL1, anti-MPR antibodies did not cause the internalization of CCR5. Second, antibodies targeting MPR is less likely to interrupt the natural function of CCR5, whereas inducing antibodies against N-terminus and ECL2 may abolish the physiological function of CCR5 (Figure 2F). The positive-charged natural ligand of CCR5 interacted with negatively charged N-terminus rather than MPR region, which also carried positive charge (30). Moreover, MPR locates far from ECL2 and is spatially distinct from the N-terminus, and the key residues in MPR face to the outer space of CCR5 rather than the binding pocket of Maraviroc (Figure 4) (7). This binding pocket might also be the binding site of natural ligand, according to a recent structure of CXCR4—chemokine complex (58). Retaining the amount and physiological function of CCR5 is of great importance for a safe vaccination since CCR5 deletion has been associated with the increased susceptibility to several pathogens (19–21). Third, HIV-blocking MPR antibodies might be induced by a linear epitope based on our immunogen design. In contrast, many HIV-blocking antibodies against N-terminus and ECL2 targeted to a conformational epitope (17, 59). A linear epitope could greatly simplify the vaccine design, whereas conformational epitopes are always a challenge.

Our study provided the evidence that antibodies against MPR could be induced in animals, and these MPR-specific antibodies were capable to block the infection of HIV-1. However, as other well-known MPRs including membrane proximal-external region of HIV-1 gp41 and the “Stalk” domain of influenza hemagglutinin (60, 61), MPR of CCR5 is poorly immunogenic since four vaccinations with Freund’s adjuvant only induced a modest titer of MPR-specific antibody. Nevertheless, total IgGs isolated from immunized animals, which might contain only a small portion of MPR-specific antibodies, could still significantly block the HIV-1 infection, demonstrating the feasibility of this approach. However, it should be noticed that Freund’s adjuvant could not be used in human, and the immunization strategy used in this study is not readily transferable to clinic. Therefore, the enrollment of a safer and more potent vaccine adjuvant (62) and delivery system (63) concomitant with an optimized immunogen design is needed to fully realize MPR’s potential for inducing potent HIV-1 blocking antibodies.

## Ethics Statement

Animal studies were carried out in accordance with the Guide for Animal Care and Use of Tsinghua University. All procedures were approved by the Committee on the Ethics and Welfare of Experimental Animals of Tsinghua University.

## Author Contributions

Y-HC and JW have designed and supervised the research. YT, PT, JYW, LZ, JL, YY, and JW performed the experiments. JW wrote the manuscript.

## Conflict of Interest Statement

The authors declare that the research was conducted in the absence of any commercial or financial relationships that could be construed as a potential conflict of interest.

## Footnotes

^1^http://sysbio.unl.edu/SVMTriP/.

^2^http://ailab.ist.psu.edu/bcpred/predict.html.

^3^http://imed.med.ucm.es/Tools/antigenic.pl.
